# Crop Rotational Effects on Yield Formation in Current Sugar Beet Production – Results From a Farm Survey and Field Trials

**DOI:** 10.3389/fpls.2018.00231

**Published:** 2018-03-01

**Authors:** Heinz-Josef Koch, Kerrin Trimpler, Anna Jacobs, Nicol Stockfisch

**Affiliations:** ^1^Institute of Sugar Beet Research (IfZ), Göttingen, Germany; ^2^Institute of Climate-Smart Agriculture, Thünen Institute, Braunschweig, Germany

**Keywords:** crop rotation, cropping interval, preceding crop, nitrogen, sugar yield

## Abstract

In Europe, the framework for sugar beet (*Beta vulgaris* L.) production was subject to considerable changes and for the future it is expected that sugar beet cultivation might concentrate around the sugar factories for economic reasons. Based on data from a national sugar beet farmers’ survey and multi-year crop rotation trials, the effects of cropping interval (number of years in between two subsequent sugar beet crops) and of preceding crops on sugar yield were elucidated under current Central European management conditions. The dominating sugar beet cropping interval was ≥4 years in the farm survey with pronounced differences between regions. However, the cropping intervals 2, 3, and ≥4 years did not affect the sugar yield. Therefore, significant differences in sugar yield between regions were assumed to be caused by multiple interactions between year, site, and farmers’ skills. Throughout Germany, the dominating preceding crops in sugar beet cultivation were winter wheat (*Triticum aestivum* L.) and winter barley (*Hordeum vulgare* L.). In the field trials, the sugar yield was 5% higher after pea (*Pisum sativum* L.) compared to maize (*Zea mays* L.) as preceding crop, while differences between the preceding crops pea and winter wheat, and wheat and maize were not significant. Repeated measurements of canopy development and leaf color during the growing season revealed a higher N-availability after pea as preceding crop. However, decreased growth after maize was not completely compensated for by high N-fertilizer doses. Overall, the causes for the differences in sugar yield between the preceding crops remained open. The results do not support concerns about substantial yield losses in sugar beet production due to a reduction in the cropping interval from 3 to 2 years. Nevertheless, short rotations with maize and sugar beet might increase the risk of *Rhizoctonia solani* crown and root rot infestation. Leguminous crops such as pea offer the potential for higher sugar beet yield with lower N-fertilizer doses.

## Introduction

Negative impacts of extended use of pesticides have fostered public criticism and the need for alternative measures to control weeds, pests, and diseases in agricultural crop production while simultaneously a growing world population has to be fed. In order to meet both goals, the concept of ecological intensification was developed (Godfray et al., 2010; Petersen and Snapp, 2015). Cultivation of annual crops in well-designed sequence with other species instead of continuous cropping or short rotations can help to control specific pathogens in arable crops and reduce the need for pesticide use to ensure high and stable yields (Coulter et al., 2011). Frequent cultivation of sugar beet (*Beta vulgaris* L.) on one field is known to stimulate infestation by soil-borne pests and diseases such as beet cyst nematode (*Heterodera schachtii* Schmidt) or black root rot (*Aphanomyces cochlioides* Drechsler) which can cause substantial yield losses (Schäufele and Winner, 1989; Hauer et al., 2016). To minimize such negative impacts, sugar beet is traditionally not grown in monoculture but in rotations with cropping intervals, here defined as the number of years in between two subsequent beet crops on the same field, of two or more years (Märländer et al., 2003).

In addition to phytopathological aspects, which are often linked to the survival of pests on crop residues, crop rotational effects are known to include a wide range of impacts related to e.g., nutrients supplied by the direct and/or previous preceding crops (Smith et al., 2008). Further, soil structural conditions affected by the rooting properties of the preceding crops plus measures taken to manage the preceding crops (soil tillage and machinery use) contribute to preceding crop effects (Ball et al., 2005; Munkholm et al., 2013). Bennett et al. (2012) reported various examples for yield responses of crops grown in short rotation or monoculture compared to diverse rotations and identified numerous biotic and abiotic factors as potential causing agents for the yield decline observed in short rotations/monoculture. Nevertheless, they also stated that evidence for the precise contribution of single factors or factor combinations is often missing due to the complexity of field experiments, but need to be clarified in future research. Finally it is worth to mention that residues from herbicides applied to a preceding crop can cause injury to a future crop (Stipičević et al., 2015; Cornelius and Bradley, 2017) and thus, may contribute to crop rotational effects.

Concerning sugar beet, current knowledge on crop rotational effects derives from field trials conducted in the 1960s up to the 1980s in Northwest and Central Europe as comprehensively summarized by Götze (2017). More recently, Götze et al. (2017) reported crop rotation effects on the stability of beet yield and quality. In these studies, almost all experimental sites were characterized by a moderate to high beet cyst nematode infestation level and the cultivation of a susceptible beet variety. Such a combination does, however, not match the current situation in agricultural practice, because choosing a beet cyst nematode tolerant or resistant variety would be highly preferable under infested conditions; tolerant and resistant varieties respond with substantially lower yield decline compared to susceptible varieties (Hauer et al., 2016). Only Draycott et al. (1978) and Liste et al. (1990) evaluated crop rotation effects on sugar beet yield in a long-term trial conducted on a soil without beet cyst nematode infestation; these trials were ceased in 1976 and 1989, respectively, and thus, cropping conditions were not comparable to current sugar beet cultivation with regard to the preceding crops included and the crop management applied (variety, nutrient supply, and crop protection). Hao et al. (2001) compared two 4-year crop rotations including two times spring wheat, one legume and one sugar beet crop, with beets grown after either legume or wheat, under irrigated conditions in the continental climate of North America. Overall, very limited knowledge exists on preceding crop and cropping interval effects under current central European soil, climatic and management conditions.

In Europe, the legal and economic framework for sugar beet production was subject to considerable changes in the past decade (2005/06: reduction in minimum beet price and sugar production quota; 2016/17: abolition of price and sugar quota restrictions), resulting in a decline in the area cultivated with sugar beet after 2005 followed by a re-increase after 2015 (EUROSTAT, 2017). This increase in cropping area exclusively took place on farms located in traditional growing regions with an existing infrastructure for beet production and processing. In Germany, such changes came along with the emergence of silage maize (*Zea mays* L.) used for biogas feedstock production (Jacobs et al., 2014) and leguminous crops such as field pea (*Pisum sativum* L.) grown on ecological focus areas (DirektZahlDurchfV, 2014). Nevertheless, in 2010–2015 winter wheat (*Triticum aestivum* L.) and winter barley (*Hordeum vulgare* L.) were still the most common preceding crops for sugar beet (60 and 20%, respectively), and sugar beet were most frequently grown with cropping intervals of 2 or 3 years (32 and 26%, respectively; Trimpler and Stockfisch, 2017). But in future, sugar beet production will likely need to face periods of low beet price and thus, concentrate close to sugar factories in order to minimize transportation costs (Isermeyer et al., 2005), thereby shortening the beet cropping interval even if shorter intervals may cause lower yield.

In order to provide information on crop rotational effects on sugar beet performance under current Central European management conditions, data from a national sugar beet farmers’ survey were evaluated to answer questions concerning: (i) What is the dominating cropping interval in Germany today? and (ii) How does sugar yield respond to decreasing cropping intervals of ≥4, 3, and 2 years under soil and climatic conditions prevailing in Germany? Further, crop rotation trials were conducted on highly productive sites in Lower Saxony and Southern Bavaria, Germany, to answer the research questions: (iii) How do field pea and maize compared to winter wheat as reference preceding crop affect sugar beet yield? and (iv) Does a high compared to a low nitrogen (N) supply level for sugar beet modify preceding crop effects? Overall, our study aimed to evaluate crop rotational effects in the context of a sustainable development of sugar beet cultivation in Central Europe.

## Materials and Methods

### Farm Survey

The survey included 2148 sugar beet fields in Germany and was carried out in seven seasons (2010–2016). The data were collected through a questionnaire sent to more than 300 sugar beet farmers per year, each providing information from his biggest sugar beet field. The farms were distributed throughout all growing regions of Germany according to the area under sugar beet. Farms were randomly picked to represent a range of farm and field sizes, crop rotations and specialties. The questionnaire provided some general information about the farm and the management practices concerning the largest sugar beet field. These included crop rotations, pesticide use, mineral and organic fertilization, sowing and harvest dates, and taproot yield plus sugar content of sugar beet, which were used to calculate the sugar yield (Trimpler et al., 2017). Taproot yield and sugar content were derived from the growers’ records received from the sugar factory. In order to consider fundamental differences in productivity between sites, the farmers were asked for the field evaluation index (German “Ackerzahl”; BodSchätzG, 2007) of their field which is provided by national inventories. The field evaluation index describes the soil’s quality together with natural conditions of the site. It includes soil texture, rootability, and field slope plus influences of climate and other factors, and ranges from about 20 (low quality for cropland) to 120 (highest quality).

Results are presented either for all farms in Germany or aggregated in sub-groups for the regions North (Lower Saxony and Schleswig-Holstein), East (Mecklenburg-Western Pomerania, Brandenburg, Saxony-Anhalt, Saxony, Thuringia), South (Bavaria, Hesse, Baden-Württemberg, Rhineland-Palatinate), and West (North Rhine-Westphalia) according to Trimpler et al. (2017).

### Field Trials

#### Crop Rotation Trial at Harste

In 2006, a crop rotation trial was established at Harste near Göttingen, Lower Saxony, Germany (51°36′23.5″N, 9°51′55.8″E), on silty loam Luvisol soil (IUSS Working Group WRB, 2006; topsoil 0–30 cm: clay 100 g kg^-1^, silt 760 g kg^-1^; organic C 13 g kg^-1^; pH (CaCl_2_) 7.2; Mg (CaCl_2_) 96 mg kg^-1^; P, K (CAL) 75, 122 mg kg^-1^, respectively). The climate was characterized by a 30-year (1981-2010) mean annual rainfall of 651 mm and a mean annual temperature of 9.2°C (Deutscher Wetterdienst [DWD], 2015). Beet cyst nematode infestation on the experimental field measured in spring 2005 was below 400 eggs and juveniles kg^-1^ of soil.

The field experiment included eight crop rotations, out of which three with sugar beet were included in this study: (1) winter wheat - winter wheat - white mustard (*Sinapis alba* L.) cover crop - sugar beet; (2) winter wheat - mustard cover crop - maize - sugar beet; (3) winter wheat - winter rapeseed - winter wheat - winter wheat - phacelia cover crop (*Phacelia tanacetifolia* L.) - field pea - white mustard cover crop - sugar beet. This allowed to compare the effects of winter wheat, maize, and field pea as preceding crops on subsequent sugar beet growth and yield. In the rotations (1) and (3), such effects included the impact of mustard cover crop that was grown in autumn between preceding crop harvest and subsequent sugar beet. Maize was grown either as corn (2006-2009) or silage maize (2010-2016). For sugar beet cultivated in 2011-2013, the amount of mineral N-fertilizer was varied as a second factor in doses of 0, 40(2011)/60(2012, 2013), 80/90, and 120 kg N ha^-1^, subsequently addressed as N0, N1, N2, and N3, respectively. For this purpose, the main plots (220 m^2^) were split up into four sub-plots (55 m^2^), resulting in a split-plot design with the preceding crop on main level and the N-fertilizer dose plot on sub-plot level. Each crop rotation element was present in the trial each year with three replicates arranged in complete blocks. Within replicates, six incomplete blocks with four out of the eight crop rotations were combined.

Primary soil tillage was conducted with a cultivator to 15-20 cm depth with two exceptions: (i) in autumn 2006-2009 after grain maize plots were moldboard ploughed to 15-20 cm depth after harvest to incorporate maize straw; (ii) in summer/autumn 2015 all plots were moldboard plowed to 25 cm depth. Sugar beet sowing was performed after seedbed preparation between late March and mid-April with placement of pelleted seeds in rows 45 cm apart and at 7.7 cm in-row distance. At 6-8-leaf-stage of plants in May, crops were manually singled to a final stand of approximately 23 cm in-row distance resulting in a plant population of 9-10 plants m^-2^. The sugar beet varieties cultivated were tolerant against beet necrotic yellow vein virus (“Rhizomania”) and beet cyst nematodes: Lucata (2007-2008), Beretta (2009-2010), Belladonna KWS (2011-2014), Lisanna KWS (2015-2016) (Federal Plant Variety Office [FPVO], 2017). The mustard cover crop grown was beet cyst nematode resistant and non-winter hard. Crop management including pesticide use followed the recommendations of the regional extension service of the federal state of Lower Saxony partially adapted according to the personal experience of the technician responsible for the trial. Weeds and leaf spot diseases were effectively controlled by pesticides. Main crop and cover crop residues were left in the field.

For all crops, the mineral N-fertilizer dose was derived according to the concept of a mineral N target value (“Sollwert”), taking into account (i) anticipated differences in N-mineralization due to the specific preceding crop and cover crop cultivation (140 kg N ha^-1^ after pea and wheat; 160 kg N ha^-1^ after maize), and (ii) the soil mineral N-content (N_min_, 0-90 cm depth) measured in March each year (Landwirtschaftskammer Niedersachsen-Geschäftsbereich Landwirtschaft, 2010). The N-fertilizer doses applied to sugar beet ranged across years between 0-85 kg N ha^-1^ (mean 45 kg N ha^-1^), 60-100 kg N ha^-1^ (mean 75 kg N ha^-1^) and 90-135 kg N ha^-1^ (mean 112 kg N ha^-1^) after pea, wheat and maize, respectively. The N-fertilizer was broadcasted immediately after sowing either as calcium-ammonium-nitrate granules (2011-2013) or ammonium-nitrate-urea solution. Cover crops were supplied with 50 kg N ha^-1^.

In addition to the March sampling date, N_min_ was determined in the N0 plots in May and June 2011-2013. Seven cores per plot were mixed to a composite sample. Soil N_min_ (NH_4_^+^ and NO_3_^-^) was extracted from a sub-sample of 100 g soil by 250 ml of 0.0125 molar CaCl_2_ solution and analyzed colorimetrically with a Continuous-Flow-Analyzer (Skalar Analytical BV, SFAS 5100, Netherlands).

In 2007-2010 and 2014-2016, sugar beet yield was determined at the end of September on a core area of 12.9 m^2^ per plot (2 adjacent rows each 14 m long) by an experimental sugar beet harvester after manual topping. In 2011-2013 the harvest plot size was 10.8 m^2^ (4 rows 6 m long). In the lab, beet taproots were washed to determine beet fresh weight, and processed to brei, out of which a sub-sample was shock-frozen and stored at -18°C until sugar analysis according to International Commission for Uniform Methods of Sugar Analysis [ICUMSA] (2007). Sugar yield was calculated from taproot yield and sugar content.

In 2011-2013, early growth of sugar beet was established by harvesting the surplus plants removed at singling to the final stand in May. On a surface of 8.8 m^2^ per N-fertilizer sub-plot entire plants (excluding fibrous roots) were manually removed from the soil by hand, counted, washed in the lab and dried to constant weight at 105°C. Dry matter yield per plant was used to calculate dry matter yield per ha at a plant population of 9 plants m^-2^. Around mid-June and mid-July leaf area index (LAI) was measured in N-fertilizer sub-plots with the LAI-2200 (LI-COR, Lincoln, NE, United States) according to the protocol of Röver and Koch (1995). The concentration of chlorophyll in young, almost fully expanded sugar beet leaves was determined with the Yara N-Tester (YARA, Germany), which operates similar to the SPAD meter (MINOLTA, Japan).

#### Crop Rotation Trial at Aiterhofen

In 2010, a crop rotation trial was established at Aiterhofen near Straubing, Bavaria (48°51′06.5″N, 12°37′58.5″E) on silty loam Luvisol soil [IUSS Working Group WRB, 2006; topsoil 0-45 cm: clay 667 g kg^-1^, silt 76 g kg^-1^; organic C 10 g kg^-1^; pH (CaCl_2_) 7.3; Mg (CaCl_2_) 106 mg kg^-1^; P, K (CAL) 172, 134 mg kg^-1^, respectively]. The climate of this site was characterized by a 30-year (1981-2010) mean annual rainfall of 757 mm and a temperature of 8.6°C (Deutscher Wetterdienst [DWD], 2015).

This experiment included four crop rotations, out of which two were included in this study: (1) winter wheat - winter wheat - white mustard cover crop - sugar beet; (2) winter wheat - white mustard cover crop - silage maize - sugar beet for comparing the effects of winter wheat and silage maize as preceding crops on subsequent sugar beet. Each crop rotation element was present in the trial each year with four replicates arranged in complete blocks (plot size 420 m^2^). Primary tillage was performed as conservation tillage in autumn, using a cultivator at a soil depth of 18 cm. For seedbed preparation in spring a rotary harrow was used. Sugar beet sowing date varied between mid-March and mid-April among years (row width 50 cm, 6 cm in-row distance). In May crops were manually singled to a final stand of approximately 24 cm in-row distance resulting in a plant population of 9 plants m^-2^.

The sugar beet varieties grown were Rhizomania tolerant and beet cyst nematode tolerant: Deborah KWS (2011-2014), Isabella KWS (2015) (Federal Plant Variety Office [FPVO], 2017). The N-fertilizer doses for sugar beet were uniform among preceding crops within each year, but varied among years from 100 to 135 kg N ha^-1^. Beet cyst nematode resistant white mustard cover crop grown after wheat harvest was supplied with 40 kg N ha^-1^. For all crops N-fertilizer was sprayed shortly before or after sowing as ammonium-nitrate-urea solution. Crop management including pesticide use followed the recommendations of the regional extension service of the federal state of Bavaria partially modified according to the personal experience of the technician responsible for the trial. Main crop and catch crop residues were left in the field.

In 2011-2015, sugar beet yield was manually determined around mid-October on a core area of 12 m^2^ per plot (3 adjacent rows each 8 m long). Further processing and analyses to establish the sugar yield followed the protocol as described previously (section “Crop Rotation Trial at Harste”).

### Statistical Analyses

For the analysis of sugar yield 2010-2016 from the farm survey, the model for the analysis of variance included the effects cropping interval, region and year. The unbalanced data set included 2148 observations and passed the normality tests. Therefore, a three way analysis of variance was performed with the General Linear Model procedure. The analysis of variance was repeated including the field evaluation index as covariate. The *F*-values of the main effects and their interactions were considered significant for *p* ≤ 0.001. All average values from the farm survey are presented as median values. All statistical analyses were conducted with the software package SAS Version 9.4 (SAS Institute Inc., Cary, NC, United States).

For the analysis of sugar yield in 2007-2016 of the Harste experiment, the statistical model included the following effects: preceding crop, year, their interaction (all fixed); year nested within replicate, block, plot (all random). For the evaluation of total plant dry matter yield in May, LAI, N-Tester and sugar yield data of 2011-2013 the model was: preceding crop, N-fertilizer dose (N0-N3), year, and their interactions (all fixed); interaction of preceding crop and replicate nested within year (random). Soil N_min_ data in 2011-2013 were calculated with the effects: preceding crop, year, their interaction (fixed); year nested within replicate (random). Sugar yield in 2011-2015 of the Aiterhofen experiment was evaluated with the model: preceding crop, year, its interaction (fixed); year nested within replicate (random).

Analyses of variance were conducted with the MIXED procedure after having checked the data residues for normal distribution with the UNIVARIATE procedure. If not normally distributed, data were square root transformed before analysis of variance. Comparisons of mean values were performed with Tukey’s LSD test at *p* ≤ 0.05. Tables and figures display re-transformed data when applicable.

## Results

### Cropping Intervals and Their Effects on Sugar Yield (Farm Survey)

The farm survey revealed that winter cereals, namely winter wheat or winter barley, were grown before sugar beet on more than 80% of the fields surveyed in the years 2010-2016. Winter wheat as preceding crop was cultivated on 57% of all fields, ranging between 55 and 60% throughout the years. Winter barley as preceding crop grew on 24% of the fields and varied from 22 to 26%. In order to eliminate effects from unusual preceding crops on sugar yield, the following analysis focused on sugar beet fields with the preceding crops winter wheat or winter barley.

**FIGURE 1:**
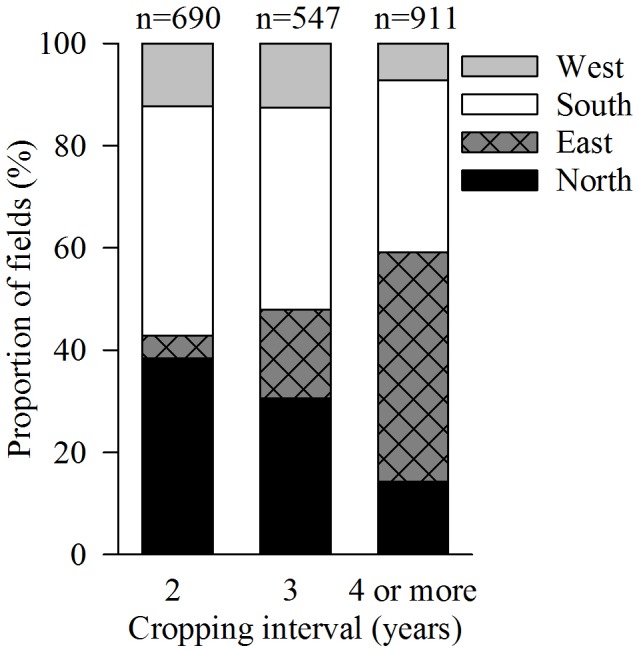
Distribution of fields with different cropping intervals throughout regions in Germany. Only fields with the preceding crops winter wheat or winter barley selected from the farm survey of sugar beet cultivation in Germany 2010–2016 are presented here (*n* = 2148).

The sugar beet cropping interval dominating was ≥4 years (**Figure 1**). In the region North, however, the most frequent cropping interval for almost half of all fields was 2 years (not shown). In contrast, over 70% of all fields in the region East showed cropping intervals of ≥4 years. The cropping intervals in the regions West and South were more evenly distributed.

The median sugar yield in the survey was 13.8 Mg ha^-1^. The variables region and year had a significant influence on the sugar yield (*p* ≤ 0.001), which ranged between 12 and 15 Mg ha^-1^ in the regions North, East, South, and West (**Table 1**, year not shown). No significant effects were observed for the variable cropping interval or the interactions between cropping interval and year, cropping interval and region and cropping interval, region and year (not shown). Across regions, increasing the cropping interval from 2 to 3 years or ≥4 years did not increase the sugar yield. It tended to decrease from cropping interval 2 to 3 years in regions North and South, while in regions East and West a slight increase occurred with increasing cropping interval.

**Table 1:** Median of sugar yield (*n* = 2148) and field evaluation index (*n* = 2121) according to sugar beet cropping intervals and regions in Germany.

Cropping interval (years)	2	3	≥4	All cropping intervals
	**Sugar yield (Mg ha^-1^)**			
	
Average	14.4	13.9	13.4	13.8
Regions:				
North	14.2	13.4	13.8	13.9
East	12.0	12.3	12.4	12.4
South	14.4	14.2	14.4	14.4
West	14.4	15.6	15.1	14.9

	**Field evaluation index**			
	
Average	73	70	62	69
Regions:				
North	78	68	55	72
East	67	72	57	60
South	70	68	66	68
West	75	75	70	73


**Only fields with the preceding crops winter wheat or winter barley were selected from the farm survey sugar beet cultivation Germany 2010–2016*.*

Subsequently, the data set was examined for influences of the field evaluation index on sugar yield (**Table 1**). The average field evaluation index was 69 and ranged from 73 to 60 between regions. All regions showed lower field evaluation indices for fields with cropping intervals of ≥4 years compared to fields with cropping intervals of 2 or 3 years. Consequently a correlation between field evaluation index and sugar yield was assumed and the field evaluation index was included as covariate in the analysis of variance. The field evaluation index turned out to be a significant covariate (*p* < 0.001) but neither the variable cropping interval nor the interaction of cropping interval with field evaluation index were significant (not shown).

### Preceding Crop Effects on Yield Formation (Field Trials)

**FIGURE 2:**
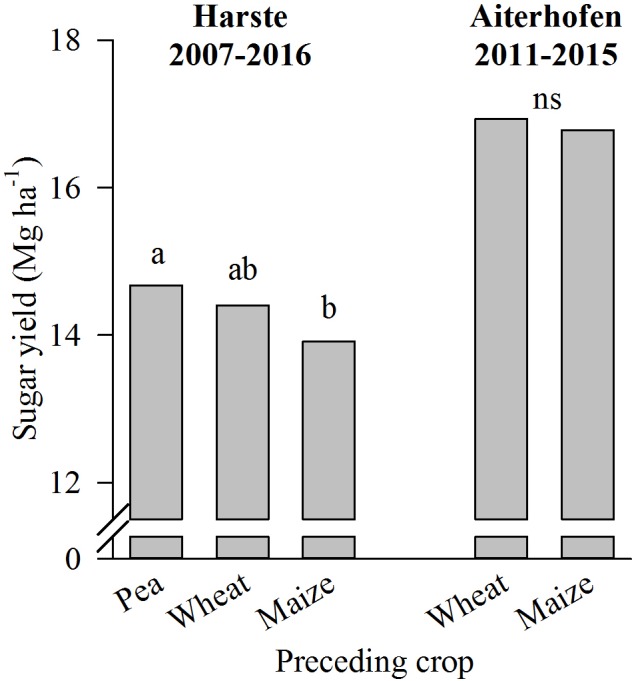
Preceding crop effect on sugar yield in the crop rotation trials at Harste in 2007–2016 and Aiterhofen in 2011–2015 (mean of field replicates and years). Different letters indicate significant differences at *p* ≤ 0.05 according to Tukey’s LSD test, ns = not significant.

At Harste, the sugar yield was significantly affected by the factors year (not shown) and preceding crop: sugar yield was higher after pea compared to maize, and intermediate after wheat as preceding crop (**Figure 2**). Similarly, the yield was not different after wheat compared to maize at Aiterhofen, and the effect of the year was significant (not shown). The interaction between year and preceding crop was not significant at Harste, but significant at Aiterhofen (not shown). Nevertheless, there was not a single year in which sugar yield was significantly different after the two preceding crops tested here (not shown). Differences in sugar yield were primarily due to differences in taproot yield and not sugar content at both sites (not shown).

**FIGURE 3:**
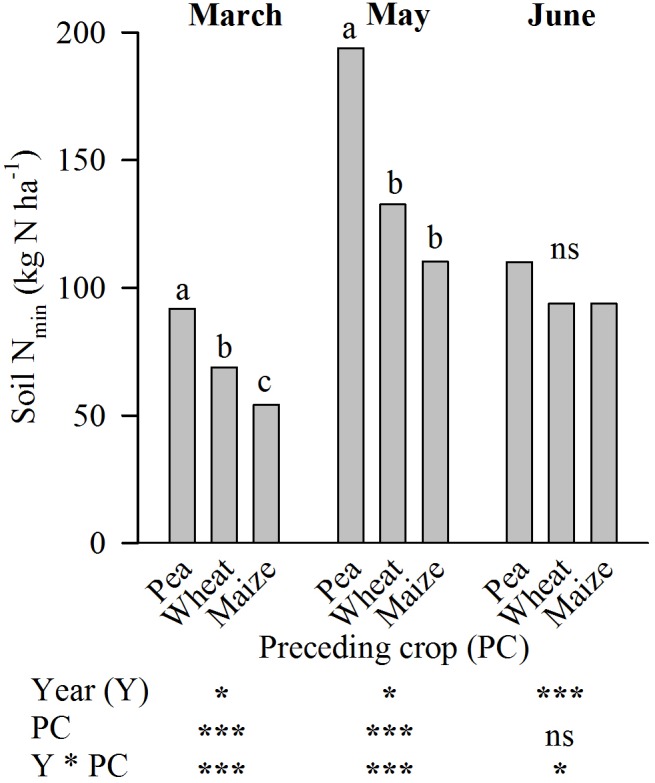
Preceding crop effect on the soil mineral N-content (N_min_, 0–90 cm soil depth) at three dates in the crop rotation trial at Harste (mean of field replicates and years 2011–2013). Different letters indicate significant differences at *p* ≤ 0.05 according to Tukey’s LSD test for individual sampling dates, ns = not significant. The table below the figure shows the results of the analysis of variance. Significance of *F*-values was displayed with ^∗^*p* < 0.05 and ^∗∗∗^*p* < 0.001.

On average of the years 2011-2013 with altered N-fertilizer treatments at Harste, the soil N_min_ in the unfertilized plots doubled from March to May and decreased again until June in all treatments (**Figure 3**). For all sampling dates, the interaction of the factors year and preceding crop was significant. This was due to significant differences among the preceding crops in 2011 and 2013, but a lack of effect in 2012 (not shown). Thus, the significantly higher mean value after pea compared to wheat and wheat compared to maize (March), and pea compared to wheat and maize (May) was due to differences occurring in 2011 and 2013 (**Figure 3**). At sampling in June, the slightly higher 3-year average of soil N_min_ in unfertilized sugar beet plots after pea was caused by a significantly higher value in 2013 only.

**FIGURE 4:**
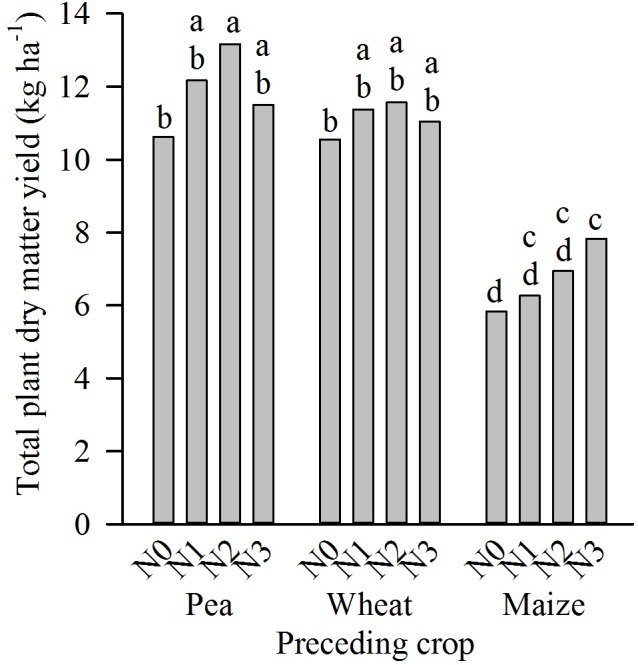
Interaction between preceding crop and N-fertilizer dose (N0–N3, for details c.f. section “Crop Rotation Trial at Harste”) on total plant dry matter yield in May in the crop rotation trial at Harste (mean of field replicates and years 2011–2013). Different letters indicate significant differences at *p* ≤ 0.05 according to Tukey’s LSD test.

Total plant dry matter yield in May was significantly affected by all main effects and interactions (**Table 2**). The interaction of year, preceding crop and N-dose was significant due to a strong yield increase with increasing N-dose after maize in 2011, while in the other combinations of year and preceding crop the N-dose had no significant effect (not shown). Across years, there was a yield increase from N0 to N2 after pea (significant) and wheat (not significant) as preceding crops, while after maize yield increased significantly from N0 to N3 (**Figure 4**). Nevertheless, this increase compensated just incompletely for the lower yield after maize compared to the other preceding crops across all N-fertilizer levels; such differences among preceding crops occurred in 2011 and 2012, but not in 2013 (not shown).

**Table 2:** Significance of *F*-values for the effects of year (2011-2013), preceding crop (pea, wheat, maize), N-fertilizer dose (N0-N3, for details c.f. section “Crop Rotation Trial at Harste”) and its interactions on parameters of sugar beet growth measured during the growing season and sugar yield in autumn in the crop rotation trial at Harste (^∗^*p* < 0.05, ^∗∗^*p* < 0.01, and ^∗∗∗^*p* < 0.001), DF = degrees of freedom.

Effect	*DF*	Total plant dry matter yield May	LAI June	LAI July	N-Tester June	N-Tester July	Sugar yield autumn
Year (Y)	2	^∗∗∗^	^∗∗∗^	^∗∗∗^	^∗∗^	^∗∗^	^∗∗∗^
preceding crop (PC)	2	^∗∗∗^	^∗∗∗^	^∗∗∗^	^∗∗∗^	^∗∗∗^	^∗∗∗^
N-dose (N)	3	^∗∗∗^	^∗∗∗^	^∗∗∗^	^∗∗^	^∗∗∗^	^∗∗∗^
Y^∗^PC	4	^∗∗∗^	^∗∗∗^	^∗^	ns	ns	^∗∗∗^
Y^∗^N	6	^∗∗^	^∗∗∗^	ns	^∗∗∗^	^∗∗∗^	^∗∗^
PC^∗^N	6	^∗^	ns	ns	ns	ns	^∗∗∗^
Y^∗^PC^∗^N	12	^∗∗^	ns	ns	ns	ns	^∗^


**FIGURE 5:**
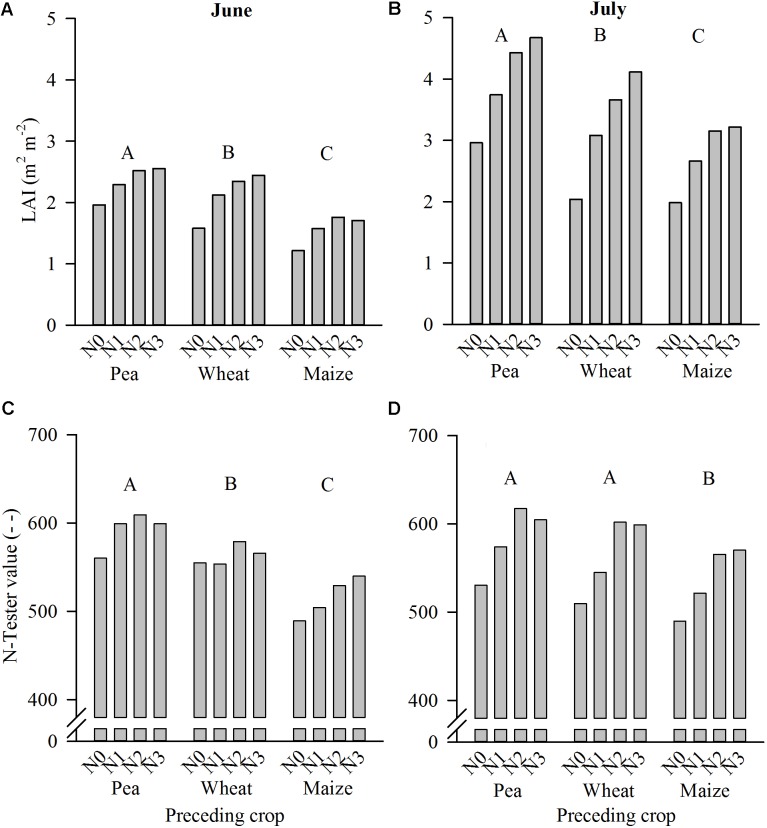
Interaction between preceding crop and N-fertilizer dose (N0–N3, for details c.f. section “Crop Rotation Trial at Harste”) on leaf area index (LAI; **A,B**) and N-Tester value **(C,D)** in June and July in the crop rotation trial at Harste (mean of field replicates and years 2011–2013). Different capital letters above column groups indicate significant differences between preceding crops across N-fertilizer doses and years at *p* ≤ 0.05 according to Tukey’s LSD test.

The development of the sugar beet canopy, measured as the LAI, and the N-supply of sugar beet leaves, measured as the N-Tester value, in June and July were significantly affected by the factors year, preceding crop and N-fertilizer dose (**Table 2**). In addition, the interaction of year and preceding crop (LAI June and LAI July) was significant, which was due to a higher LAI value after pea compared to wheat and maize in 2011, while in 2012 and 2013 LAI was equal after pea and wheat but higher compared to maize (not shown). Further, year and N-dose interacted significantly for LAI June, N-Tester June, and N-Tester July (**Table 2**). For LAI June, N3 caused higher values compared to N0 in 2011; in 2012 the difference between N2 and N0 was significant, and in 2013 values of N1 to N3 were higher than those of N0 (not shown). In June, N1 to N3 caused significantly higher N-Tester values than N0 in 2011 while in 2012 a similar but insignificant trend as in 2011 was obvious, and in 2013 the N-dose had no effect on the N-Tester values (not shown). In July, the N-Tester value of N-fertilizer dose N1 was significantly higher than of N0, and higher with N3 compared to N2; in 2012 only, N0 caused significantly lower values compared to N1-N3, and in 2013 the N-dose had no effect on the N-Tester value in July (not shown). Overall, despite such manifold interactions the N-doses N1 and N2 caused a substantial increase in LAI and N-Tester value at both measuring dates compared to the respective lower N-dose, while N3 did not further increase values compared to N2 in most combinations of year and preceding crop, and as the mean across years and preceding crops. Further, across years and N-fertilizer doses LAI and N-Tester values were highest after pea, intermediate after wheat and lowest after maize (**Figure 5**).

**FIGURE 6:**
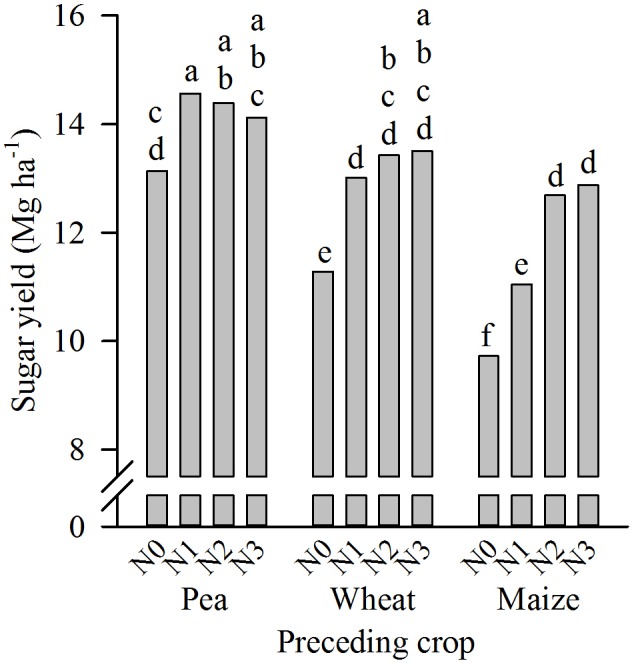
Interaction between preceding crop and N-fertilizer dose (N0–N3, for details c.f. section “Crop Rotation Trial at Harste”) on sugar yield in autumn in the crop rotation trial at Harste (mean of field replicates and years 2011–2013). Different letters indicate significant differences at *p* ≤ 0.05 according to Tukey’s LSD test.

Sugar yield in autumn was significantly affected by all main effects and interactions (**Table 2**). The interaction of year, preceding crop and N-dose was due to a significant yield increase from N0 to N2 (and N3) after maize in each year of the study period, while after pea, the N-fertilizer dose did not reveal a significant effect in any year (not shown). In contrast, after wheat as preceding crop increasing the N-dose increased the sugar yield in 2011 and 2013 (N0-N2), but not in 2012 (not shown). On average across years, increasing the N-dose from N0 to N1 significantly increased sugar yield after all preceding crops. However, after maize only, a further yield increase was obtained when increasing the N-dose from N1 to N2 (**Figure 6**); increasing the N-dose from N2 to N3 caused no further yield increase and even at the highest N-dose yield remained lower after maize compared to pea.

## Discussion

Future development of the legal and economic framework in Europe for sugar beet production and crop production in general might cause considerable changes in crop rotations with sugar beet. Farm survey data and results from crop rotation trials were analyzed for effects of cropping intervals, preceding crops, and the potential of elevated mineral N-supply for leveling out the yield decline observed after maize as preceding crop.

Data from the farm survey revealed the current situation concerning preceding crops and cropping intervals in sugar beet cultivation in Germany. We expected a major effect on sugar beet yield by the specific crop that was grown directly prior to sugar beet. Therefore, we concentrated our evaluation of cropping interval effects on fields with winter wheat or winter barley before sugar beet, being the most frequent preceding crops before sugar beet in Germany. Simultaneously, these crops were represented in the farm survey with a frequency high enough to allow for the formation of sub-groups such as preceding crop intervals. Further, sugar beet growth and yield is the result of a multitude of impacts including genetic, environmental, and management factors (van Ittersum et al., 2013). Thus, the yield produced on any individual field presented in our farm survey data set is the result of a specific combination of such influencing factors. In addition, factors such as weather conditions, soil properties, pest and disease occurrence might differ regionally and correlate with the cropping interval. Therefore, we evaluated cropping interval effects within regions and anticipated a yield increase from 2 to 3 and 3 to ≥4 years of cropping interval. This expectation, however, was not confirmed neither for the nationwide data nor for the four regional data sets. In addition, the lack of difference in sugar yield between 2 to ≥4 years cropping intervals in the farm survey data was not attributed to a bias with soil fertility as assessed by including the field evaluation index as covariate in the statistical analyses.

Further, the farm survey showed that differences in sugar yield between regions were larger than differences between cropping intervals. A significant influence of the year was determined in a previous evaluation of sugar yield data from the farm survey for the years 2010-2014 (Trimpler et al., 2017). By means of a principal component analysis, the combination of site (soil type and field evaluation index), weather (year), and management specific (N-fertilization, pesticide use intensity) variables proved to influence the sugar yield significantly. Nevertheless, only 37% of the variance of the data could be explained by these variables, underlining the complexity of influencing factors and their interactions for cropping systems in real farm situations as stated by Bennett et al. (2012).

The lack of a significant effect of the cropping interval of 2, 3, and ≥4 years in our farm survey data is presumably explainable by the increased use of nematode tolerant varieties. Tolerance describes a limited yield decline compared to a susceptible plant (Müller, 1989). In 2011, up to 30% of all sugar beet varieties grown by farmers had a tolerance toward beet cyst nematode infestation in some regions (Buhre et al., 2014). Until 2016, the proportion of nematode tolerant varieties steadily increased to more than 30% of the whole sugar beet area in Germany. It is further supposed that nematode tolerant varieties are preferably grown on fields with an elevated beet cyst nematode infestation. For those fields, one cause for a reduced sugar yield in short rotations is eliminated. Besides, nematodes are known to reduce root yield depending on the environmental situation (temperature and water availability) of the particular growing season (Hauer et al., 2016), which causes additional variation in the yield of sugar beet grown on nematode infested fields.

The cropping interval effects obtained from the farm survey data are confirmed by field trial results, although information on cropping interval effects is available only from older field trials, which just partly reflect the conditions of current sugar beet cultivation. In a long-term trial located in Central Germany, Liste et al. (1990) found no difference in taproot yield among 1, 2, and 3 years cropping intervals on a site without beet cyst nematode infestation, while the 0 year cropping interval caused 14% yield decline, and 4 years of cropping interval increased taproot yield by 4% compared to 1-3 years cropping intervals. In contrast, at another site highly infested with beet cyst nematodes the yield of the nematode susceptible variety cultivated continuously increased by 35% from 0 to 4 years of cropping interval on average from 1974 to 1989 (Liste et al., 1990). However, the yield increase due to increasing cropping intervals in the relevant range of 2-3 and 3-4 years accounted for an increase in yield of less than 4 and 7%, respectively. Deumelandt et al. (2010) found a white sugar yield increase of 2 and 6% for the same type of comparison in the same trial but for the years 1991-2006, while Götze et al. (2017) reported an increase of 7 and 1% on average of the years 2002-2016. Overall, the size of the yield increase due to increasing the cropping interval from 2 to 4 years was relatively low even under beet cyst nematode infested conditions.

In addition to cropping interval effects, crop rotational effects mainly derive from the influence of the immediate preceding crop on the growth of the subsequent one (Hao et al., 2001). In our field trials conducted on highly productive sites, sugar yield in autumn was significantly higher by about 5% after pea compared to maize as preceding crop at Harste, while differences between the preceding crops pea and winter wheat (Harste), or wheat and maize were only small (Aiterhofen). Nevertheless, sugar yield tended to be higher after wheat than after maize at both sites in the long-term average. Regarding the higher yield of sugar beet after pea compared to maize at Harste it has to be acknowledged that the cropping interval for sugar beet simultaneously differed with 5 years in the rotation with pea compared to 2 years in the maize rotation. Thus, we cannot exclude that the wider cropping interval might have contributed to the higher yield after pea. However, taking into account the lack of cropping interval effects in the farm survey data and only small effects in several field trials as discussed above (Liste et al., 1990; Deumelandt et al., 2010; Götze et al., 2017) we hypothesize that the cropping interval effect was negligible at Harste.

Possible negative influences of maize as preceding crop before sugar beet may include effects of poor soil structure due to heavy machinery and frequent passes during harvest under wet soil conditions (Chamen et al., 1992). However, the machinery used in our field trials was smaller and lighter than the machines used on farmers’ fields and a severe impact on soil structure can be excluded. Nonetheless, the N-availability for sugar beet which had maize as preceding crop was obviously reduced as indicated by lower N-Tester values, which did not reach the level of those sugar beet cultivated after winter wheat or pea even at the highest N-doses given at Harste in 2011-2013. In addition, the sugar beet grown after pea yielded higher compared to maize as preceding crop in the other years of investigation, even though the N-fertilizer dose was substantially lower after pea. Therefore, we suggested that other effects than the N-availability were additionally limiting plant growth and yield performance of sugar beet grown after maize as preceding crop. These effects were variable among years, indicating that temperature and precipitation or soil moisture during spring and early summer (Kenter et al., 2006) as well as pathogens, such as *Heterodera schachtii* or *Rhizoctonia solani* Kühn (Anees et al., 2010; Hauer et al., 2016), are interacting effects ruling the conditions for sugar beet growth and yield.

**FIGURE 7:**
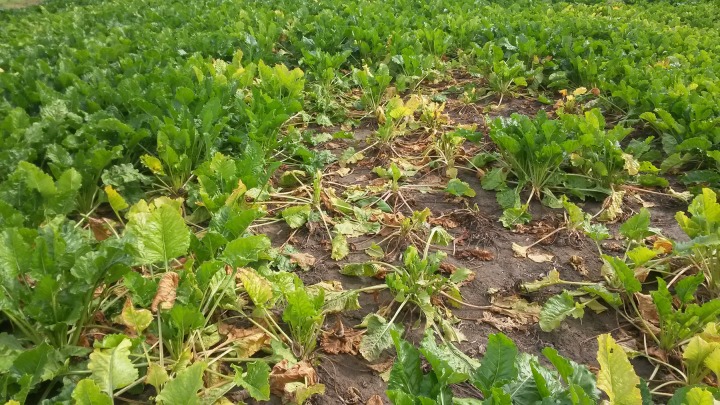
Sugar beet plant losses caused by Rhizoctonia crown and root rot infestation in the fourth cycle of the crop rotation winter wheat – white mustard cover crop – maize – sugar beet in the Harste crop rotation trial, September 01, 2017. Diseased patches occurred adjacent to healthy plants in two out of three replicate plots.

Although we did not observe any symptom of Rhizoctonia infestation in the susceptible beet variety grown in our trials in 2007-2016, we cannot exclude a low level infestation by this disease causing some sugar yield reduction when sugar beet was grown after maize. Maize is a host for the soil-borne fungus *Rhizoctonia solani*, anastomosis group 2-2IIIb, the causing agent of Rhizoctonia crown and root rot in sugar beet. In other studies, a high frequency of host crops was shown to increase infestation level (Buhre et al., 2009; Kluth and Varrelmann, 2010). At Harste, in 2017 an increasing risk of Rhizoctonia infestation became obvious in the fourth rotational cycle of the wheat - white mustard cover crop - maize - sugar beet rotation, when Rhizoctonia occurred in several sugar beet plots for the first time (**Figure 7**).

In addition, residues of the herbicides applied in maize as preceding crop before sugar beet might have caused toxic effects and thus growth reduction in the subsequent sugar beet. Although maize herbicides were chosen for high compatibility with sugar beet, elevated concentrations of terbuthylazin and desethylterbuthylazin were detected in selected topsoil samples from plots with maize compared to wheat grown before sugar beet at Harste in summer 2012 (not shown). Residual herbicide and/or Rhizoctonia effects, however, could not explain the positive impact of pea compared to wheat as preceding crop. For the rotation including pea with a 5-year cropping interval for sugar beet, one might hypothesize that the overall infestation pressure exerted by other beet specific soil-borne pests and diseases, such as *Phoma betae* and *Aphanomyces cochlioides*, was lower as when grown in 2-year intervals even if we never observed related disease symptoms. Overall, the causes for the differences in sugar yield namely between pea and maize as preceding crops remain open for the Harste trial up to date. Similarly, Bennett et al. (2012) summarize in their review of a broad range of studies in various crops that in addition to plant pathogens numerous biotic and abiotic factors were supposed as being involved in yield decline caused by cultivation in short rotations or monoculture; but due to the complex nature of cropping systems evidence for the specific significance of single factors or factor combinations is usually lacking.

The farm survey revealed that the current situation concerning preceding crops in sugar beet cultivation is rather uniform throughout Germany. Winter cereals as dominating preceding crops are supplemented by winter wheat as the succeeding crop after sugar beet on more than 75% of the fields (Trimpler and Stockfisch, 2017). Stein and Steinmann (2018) reported similar results for crop sequences including sugar beet for Lower Saxony during the years 2005-2011. Provided that a crop sequence on one field correlates with the crops grown on neighboring fields in the same year, the cropping interval for sugar beet provides an estimation of the cropping density for sugar beet within one region. If a larger acreage of sugar beet was grown within a region this could increase the disease pressure, especially leaf spot diseases and virus diseases transmitted by aphids. Decreases in yield stability or overall lower yield could result from this concentration effect (Lin, 2011) and need intensive monitoring. Contrastingly, our study demonstrates that introducing leguminous crops into cereal dominated crop rotations offers the potential for increasing the yield of subsequent sugar beet. Simultaneously, it allows for a reduced N-fertilizer input, which contributes to lower greenhouse gas emissions of sugar beet. Similar positive effects on yield and N-fertilizer requirement were reported recently when replacing sunflower by pea in a wheat - sorghum - sunflower rotation under Mediterranean climate (Plaza-Bonilla et al., 2017).

## Conclusion

For future changes in sugar beet production and steadily increasing demands on the sustainable development of crop cultivation, our data set from the farm survey did not support the expectation that shorter sugar beet cropping intervals are to cause dramatic yield losses in sugar beet. As long as growers do not violate fundamental crop rotation rules, the yield seems to rely more on the influences of year (weather) and management. A preceding crop different to the ‘classic’ winter cereal is not expected to lead to drastic changes in sugar yield as found in our field trials. Anyway, we showed a trend that pea as preceding crop offers the opportunity for gaining some yield increase with a lower amount of N-fertilizer, which may contribute to lower greenhouse gas emission of sugar beet production. Although the Harste field trial provides data from a period of 10 years, specific preceding crop effects, which have not been detected up to now, might start to appear in future. In short rotations with sugar beet and maize, Rhizoctonia infestation might become a serious threat for sugar beet production.

## Author Contributions

H-JK developed the basic concept of the field trials, conducted the respective statistical data evaluation and developed most of the figures and tables, and wrote substantial parts of the text. AJ was responsible for the coordination, data collection and evaluation of the field trials, and contributed to the interpretation of the field trial results and the text of the manuscript. NS was responsible for developing the farm survey, arrangement of the corresponding table and figure, and wrote parts of the manuscript. KT and NS analyzed the survey data including the respective statistical calculations and interpreted the results.

## Conflict of Interest Statement

The authors declare that the research was conducted in the absence of any commercial or financial relationships that could be construed as a potential conflict of interest.
